# Supplementary material for: Two Novel Schiff Base Manganese Complexes as Bifunctional Electrocatalysts for CO2 Reduction and Water Oxidation
Source: Molecules. 2023 Jan 20;28(3):1074. doi: 10.3390/molecules28031074 (PMC9920694; doi:10.3390/molecules28031074)
Supplement: Supplementary file 1 [file molecules-28-01074-s001.zip › molecules-2166486-supplementary.pdf]

SUPPORTING INFORMATION:

Two Novel Schiff Base Manganese Complexes as Bifunctional Electrocatalysts for CO_2 Reduction and Water Oxidation

Xin Zhao[1], Jingjing Li [2], Hengxin Jian [1], Mengyu Lu [1], and Mei wang [1*]

Contents **Page No**

Scheme A1. The synthesis scheme for H_2L and the metal complexes 1 and 2 under study. 2

Figure A1. IR spectrum of the complex 1. 2

Figure A2. IR spectrum of the complex 2. 3

Figure A3. The magnetic moment (μ_{eff}) of complex 1. 3

Figure A4. In situ UV-vis spectra of complex 1 in DMF:H_2O (3:7 v/v) solution containing 0.2 M phosphonate buffer solution at 1.27 V (a) and 1.58 V (b), and in situ UV-vis spectra of complex 2 in DMF:H_2O (3:7 v/v) solution containing 0.2 M phosphonate buffer at 1.15 V (c). 4

Figure A5. Cyclic voltammograms of different concentrations of 0.5 mM complex 1 (a) and 2 (b) in DMF:H_2O (3:7 v/v) solution containing 0.2 M phosphonate buffer at optimal pH (scanning rate is 100 mV s^{-1}). The inset is the relationships of catalytic currents with concentrations. 4

Figure A6. Cyclic voltammetry of 1 (a) and 2 (b) in DMF under CO_2 recorded at scan rates range from 100 to 500 mV s^{-1}. The inset is the relationships of the peak currents with the square root of the scan rates. 5

Figure A7. Cyclic voltammetry of complexes 1 (a) and 2 (b) in the 0.1 M $^nBu_4NPF_6$ DMF solution under CO_2 in different concentrations (the scanning rate is 100 mV s^{-1}). The inset is the relationship between complex concentration and reduction peak current. 5

Figure A8. In situ UV-vis spectra of complex 1 in 0.1 M $^nBu_4NPF_6$ DMF at -1.65 V (a) and in situ UV-vis spectra of complex 2 in 0.1 M $^nBu_4NPF_6$ DMF solution at -1.60 V (b). 6

Table A1. Nomenclature table for all the special symbols in the text. 6

Table A2. Comparison of different parameters of the reported similar homogeneous catalysts in recent years. 7

Table A3. Crystallographic data and structure correction parameters of complexes 1 and 2. 7

Table A4. The main key length and key angle of the complex 1 and 2. 8

Table A5. Comparison of different parameters of complexes for water oxidation in recent years. 9

Table A6. Comparison of different parameters of complexes for carbon dioxide reduction in recent years. 9

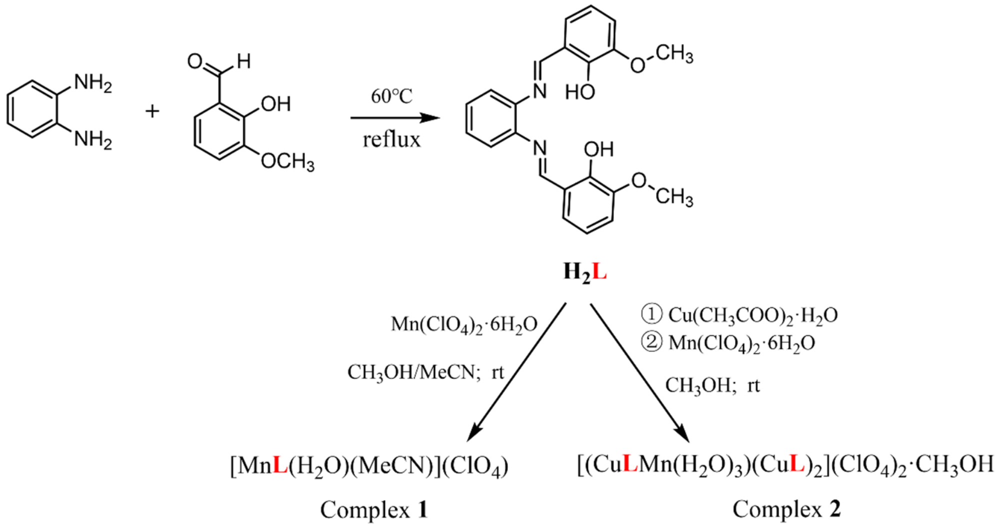

Scheme S1. The synthesis scheme for H_2**L** and the metal complexes **1** and **2** under study.

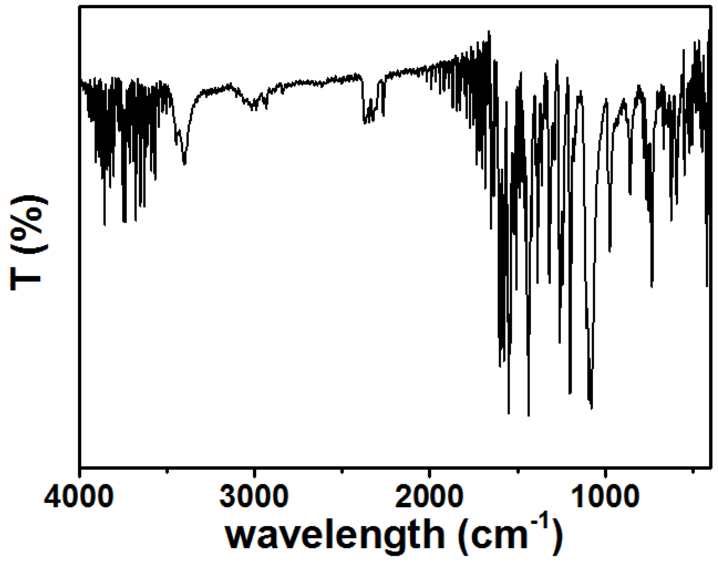

Figure S1. IR spectrum of the complex **1**.

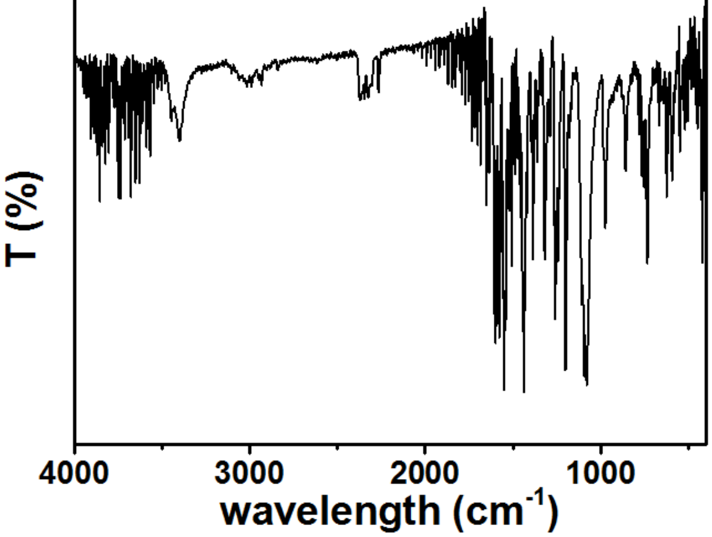

Figure S2. IR spectrum of the complex **2**.

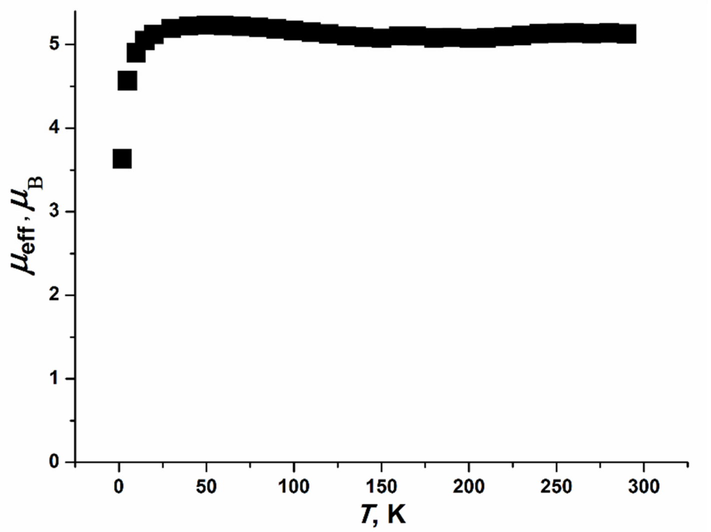

Figure S3. The magnetic moment (μ_{eff}) of complex **1**.

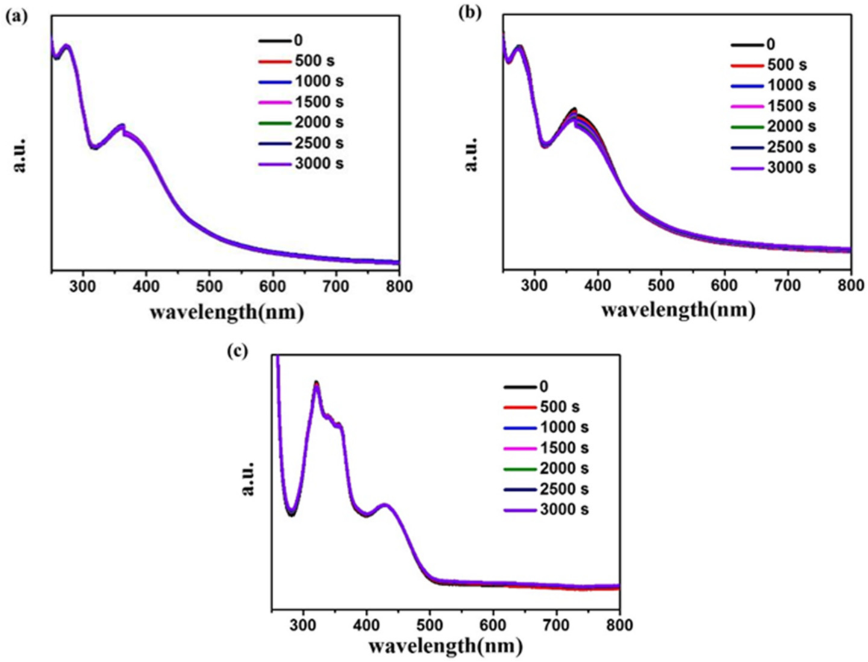

Figure S4. In situ UV-vis spectra of complex **1** in DMF:H_2O (3:7 v/v) solution containing 0.2 M phosphonate buffer solution at 1.27 V **(a)** and 1.58 V **(b)**, and in situ UV-vis spectra of complex **2** in DMF:H_2O (3:7 v/v) solution containing 0.2 M phosphonate buffer at 1.15 V **(c)**.

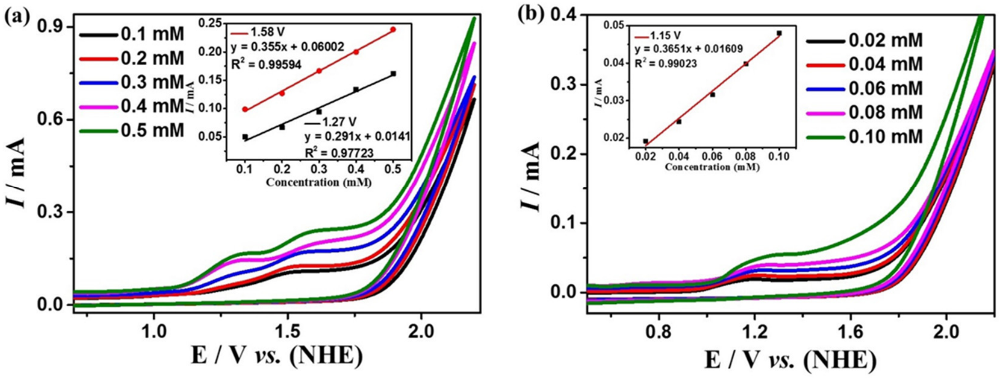

Figure S5. Cyclic voltammograms of different concentrations of 0.5 mM complex **1 (a)** and **2 (b)** in DMF:H_2O (3:7 v/v) solution containing 0.2 M phosphonate buffer at optimal pH (scanning rate is 100 mV s^{-1}). The inset is the relationships of catalytic currents with concentrations.

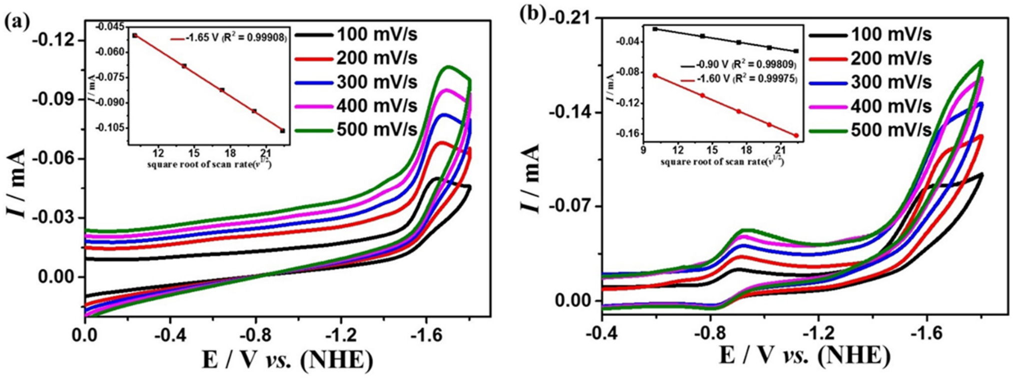

Figure S6. Cyclic voltammetry of **1 (a)** and **2 (b)** in DMF under CO_2 recorded at scan rates range from 100 to 500 mV s^{-1}. The inset is the relationships of the peak currents with the square root of the scan rates.

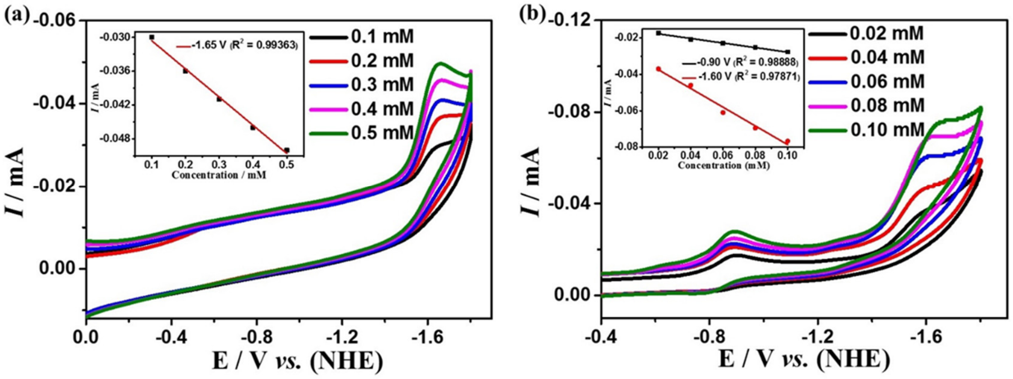

Figure S7. Cyclic voltammetry of complexes **1 (a)** and **2 (b)** in the 0.1 M $^{n}Bu_4NPF_6$ DMF solution under CO_2 in different concentrations (the scanning rate is 100 mV s^{-1}). The inset is the relationship between complex concentration and reduction peak current.

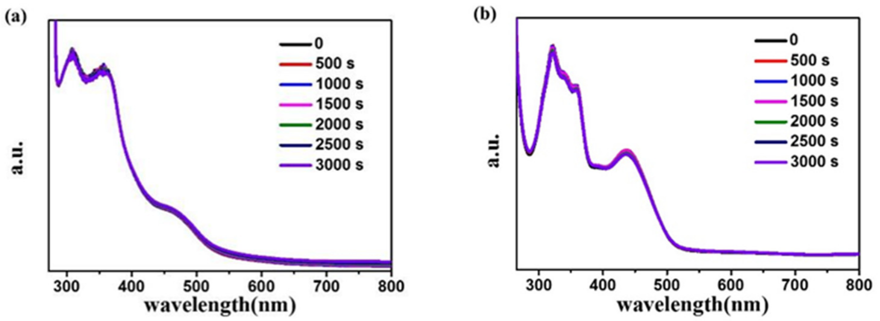

Figure S8. In situ UV-vis spectra of complex **1** in 0.1 M $^{n}Bu_4NPF_6$ DMF at -1.65 V **(a)** and in situ UV-vis spectra of complex **2** in 0.1 M $^{n}Bu_4NPF_6$ DMF solution at -1.60 V **(b)**.

Table S1. Nomenclature table for all the special symbols in the text.

Abbreviation	Name/Description
Ar	Argon
CV	cyclic voltammogram
CO_2	carbon dioxide
CPE	controlled potential electrolysis
CoFPc	perfluorinated cobalt phthalocyanine
DMF	dimethylformamide
FE	Faradaic efficiency
FTO	F-doped tin oxide
GC	glassy carbon
NHE	normal hydrogen electrode
OER	Oxygen Evolution Reaction
PCET	proton-coupled electron transfer
TOF	turnover frequency
UV-Vis	ultraviolet-visible
F	Faraday constant (96,485 C mol^{-1})
i_{cat}	catalytic current
i_p	peak current measured without substrate
n_{cat}	number of electrons involved in the catalytic reaction
n_p	number of electrons participating in the noncatalytic redox reaction (n_p = 1)
R	general gas constant (8.314 J K^{-1} mol^{-1})
T	Kelvin unit temperature
v	scan rate (0.1 V s^{-1})
η	Overpotential
μ_{eff}	magnetic moment

Table S2. Comparison of different parameters of the reported similar homogeneous catalysts in recent years.

catalyst	OER			CO_2RR			ref
	FE(%)	η(mV)	TOF(s^{-1})	FE(%)	η(mV)	TOF(s^{-1})	
$[(tpy)(Mebim\text{-}py)Ru^{II}(S)]^{2+}$	-	-	-	85	1650	-	[1]
$[(^{Bu}tpy)Ru(phenCO_2)][PF_6]$	-	-	-	-	-	-	[2]
$[Cu^{II}L^1{}_2](ClO_4)_2{\cdot}2CH_3OH$	-	-	9.20	-	-	2.99	[3]
$(Et_3NH)_2[Co^{III}{}_2Co^{II}(OH_2)(pda)_5]$	96	233	2.22	9.1	-	0.93	[4]
$[Mn^{III}L(H_2O)(MeCN)](ClO_4)$	88	728	3.66	4	-	0.38	This work
$[(Cu^{II}L_1Mn^{II}(OH_2)_3)(Cu^{II}L_1)_2](ClO_4)_2{\cdot}CH_3OH$	92	216	7.88	48	-	15.97	This work

[2] This work only studied the catalytic capability of Ru complex, but with no any quantitative values for catalysis.

Table S3. Crystallographic data and structure correction parameters of complexes **1** and **2**.

Parameter	**1**	**2**
Chemical formula	$C_{24}H_{23}ClMnN_3O_9$	$C_{67}H_{64}Cl_2Cu_3MnN_6O_{24}$
Formula weight	587.84	1653.70
Crystal system	Monoclinic	Triclinic
space group	*P*121/*c*1	*P*-1
a (Å)	10.4754(13)	13.620(2)
b (Å)	16.4898(19)	20.403(3)
c (Å)	14.7342(16)	26.782(3)
α (°)	90	91.533(4)
β (°)	110.019(3)	91.052(6)
γ (°)	90	92.062(5)
V ($Å^3$)	2391.4(5)	7433.8(18)
Z	4	4
ρcalcd (g m^{-3})	1.633	1.478
μ (mm^{-1})	0.726	1.164
Reflections collected	9034	70702
F(000)	1208.0	3384.0
R_{int}	0.0601	0.0741
T (K)	170	170
Final R indices	R_1 = 0.0546	R_1 = 0.1179
[I > 2sigma(I)]	wR_1 = 0.1457	wR_1 = 0.3161
R indices (all data)	R_2 = 0.0814	R_2 = 0.1490
	wR_2 = 0.1729	wR_2 = 0.3345
Gof	1.135	1.041

Table S4. The main key length and key angle of the complex **1** and **2**.

bond	d. Å	bond	d. Å
		complex **1**	
Mn(01)-O(1)	2.257(2)	Mn(01)-N(2)	1.973(3)
Mn(01)-O(3)	1.863(2)	Mn(01)-N(1)	1.978(3)
Mn(01)-O(2)	1.868(2)	Mn(01)-N(3)	2.380(4)
		complex **2**	
Cu(6)-O(26)	1.900(7)	Cu(3)-O(14)	1.876(7)
Cu(6)-N(11)	1.895(9)	Cu(3)-N(6)	1.911(11)
Cu(6)-O(25)	1.876(8)	Cu(3)-N(5)	1.940(9)
Cu(6)-N(12)	1.935(9)	Cu(4)-O(16)	1.875(8)
Cu(1)-O(2)	1.918(8)	Cu(4)-N(7)	1.953(10)
Cu(1)-O(1)	1.900(8)	Cu(4)-N(8)	1.949(9)
Cu(1)-N(1)	1.950(10)	Cu(4)-O(17)	1.890(8)
Cu(1)-N(2)	1.918(9)	Mn(1)-O(5)	2.216(7)
Cu(2)-O(5)	1.840(7)	Mn(1)-O(11)	2.119(9)
Cu(2)-O(6)	1.908(7)	Mn(1)-O(6)	2.183(8)
Cu(2)-N(4)	1.899(9)	Mn(1)-O(7)	2.117(8)
Cu(2)-N(3)	1.895(10)	Mn(1)-O(9)	2.163(11)
Cu(5)-O(21)	1.867(7)	Mn(2)-O(21)	2.209(7)
Cu(5)-O(20)	1.923(8)	Mn(2)-O(00J)	2.147(8)
Cu(5)-N(9)	1.875(9)	Mn(2)-O(20)	2.142(8)
Cu(5)-N(10)	1.937(11)	Mn(2)-O(015)	2.109(9)
Cu(3)-O(12)	1.899(7)	Mn(2)-O(22)	2.132(9)

Angle	ω, deg	Angle	ω, deg
		complex **1**	
O(1)-Mn(01)-N(3)	178.05(9)	O(2)-Mn(01)-N(1)	93.18(11)
O(3)-Mn(01)-O(1)	92.21(9)	O(2)-Mn(01)-N(3)	90.85(10)
O(3)-Mn(01)-O(2)	91.37(9)	N(2)-Mn(01)-O(1)	93.00(10)
O(3)-Mn(01)-N(2)	93.05(9)	N(2)-Mn(01)-N(1)	82.35(11)
O(3)-Mn(01)-N(1)	175.29(10)	N(2)-Mn(01)-N(3)	86.91(11)
O(3)-Mn(01)-N(3)	89.74(10)	N(1)-Mn(01)-O(1)	89.05(10)
O(2)-Mn(01)-O(1)	89.09(10)	N(1)-Mn(01)-N(3)	89.01(11)
O(2)-Mn(01)-N(2)	175.03(11)		
		complex **2**	
O(26)-Cu(6)-N(12)	94.9(4)	O(14)-Cu(3)-N(5)	176.8(4)
N(11)-Cu(6)-O(26)	178.7(4)	N(6)-Cu(3)-N(5)	84.4(4)
N(11)-Cu(6)-N(12)	84.0(4)	O(16)-Cu(4)-N(7)	176.8(4)
O(25)-Cu(6)-O(26)	87.9(3)	O(16)-Cu(4)-N(8)	93.4(4)
O(25)-Cu(6)-N(11)	93.1(4)	O(16)-Cu(4)-O(17)	88.2(4)
O(25)-Cu(6)-N(12)	176.7(4)	N(8)-Cu(4)-N(7)	84.7(4)
O(2)-Cu(1)-N(1)	178.7(4)	O(17)-Cu(4)-N(7)	93.5(4)
O(1)-Cu(1)-O(2)	88.7(4)	O(17)-Cu(4)-N(8)	176.4(4)
O(1)-Cu(1)-N(1)	92.3(4)	O(11)-Mn(1)-O(5)	94.0(3)
O(1)-Cu(1)-N(2)	176.4(4)	O(11)-Mn(1)-O(6)	90.8(3)
N(2)-Cu(1)-O(2)	94.3(4)	O(11)-Mn(1)-O(9)	86.0(4)
N(2)-Cu(1)-N(1)	84.7(4)	O(6)-Mn(1)-O(5)	69.1(2)

O(5)-Cu(2)-O(6)	83.4(3)	O(7)-Mn(1)-O(5)	89.5(3)
O(5)-Cu(2)-N(4)	178.2(4)	O(7)-Mn(1)-O(11)	176.4(3)
O(5)-Cu(2)-N(3)	95.5(4)	O(7)-Mn(1)-O(6)	90.3(3)
N(4)-Cu(2)-O(6)	97.1(4)	O(7)-Mn(1)-O(9)	91.1(4)
N(3)-Cu(2)-O(6)	178.6(4)	O(9)-Mn(1)-O(5)	148.2(3)
N(3)-Cu(2)-N(4)	84.0(4)	O(9)-Mn(1)-O(6)	142.7(3)
O(21)-Cu(5)-O(20)	81.7(3)	O(00J)-Mn(2)-O(21)	92.3(3)
O(21)-Cu(5)-N(9)	176.9(3)	O(20)-Mn(2)-O(21)	69.5(3)
O(21)-Cu(5)-N(10)	97.0(4)	O(20)-Mn(2)-O(00J)	93.3(3)
O(20)-Cu(5)-N(10)	178.2(4)	O(015)-Mn(2)-O(21)	90.3(3)
N(9)-Cu(5)-O(20)	96.4(3)	O(015)-Mn(2)-O(00J)	176.5(3)
N(9)-Cu(5)-N(10)	84.8(4)	O(015)-Mn(2)-O(20)	89.8(3)
O(12)-Cu(3)-N(6)	177.5(4)	O(015)-Mn(2)-O(22)	87.8(4)
O(12)-Cu(3)-N(5)	93.6(4)	O(22)-Mn(2)-O(21)	143.8(3)
O(14)-Cu(3)-O(12)	88.5(3)	O(22)-Mn(2)-O(00J)	88.8(3)
O(14)-Cu(3)-N(6)	93.5(4)	O(22)-Mn(2)-O(20)	146.6(3)

Table S5. Comparison of different parameters of complexes for water oxidation in recent years.

catalyst	η(mV)	TOF(s^{-1})	ref
$[Cu_2(TPMAN)(\mu\text{-}OH)](CF_3SO_3)_3$	780	0.78	[5]
[Cu(I)(MePzPy)]PF_6	674	9.77	[6]
6-FP-Co-OMC-1	400	0.53	[7]
C30H38N4Rh_2Cl	325	0.8	[8]
$[(Cu^{II}L_1Mn^{II}(OH_2)_3)(Cu^{II}L_1)_2](ClO_4)_2\cdot CH_3OH$	216	7.88	This work

Table S6. Comparison of different parameters of complexes for carbon dioxide reduction in recent years.

catalyst	TOF(s^{-1})	ref
$[W(bpy\text{-}H)(CO)_4]$	0.002	[9]
NiPcP	6.43	[10]
3D-Por(Co/H)-COF	1.28	[11]
$Cr(^{tbu}dhbpy)Cl(H_2O)$	5.7	[12]
$[Ni(qlca)Cl_2]$	0.83	[13]
$(Et_3NH)_2[CoIII_2CoII(OH_2)(pda)_5]\cdot H_2O$	0.93	[4]
$[(CuIIL_1MnII(OH_2)_3)(CuIIL_1)_2](ClO_4)_2\cdot CH_3OH$	15.97	This work

References

1. Chen, Z.F.; Concepcion, J.J.; Brennaman, M.K.; Kang, P.; Norris, M.R.; Hoertz, P.G.; Meyer, T.J. Splitting CO_2 into CO and O_2 by a single catalyst. *PNAS* **2012**, 109, 15606-15611.
2. Das, B.; Ezzedinloo, L.; Bhadbhade, M.; Bucknall, M.P.; Colbran, S.B. Strategic design of a ruthenium catalyst for both CO_2 reduction and H_2O oxidation: the electronic influence of the co-ligands. *Chem. Commun*. **2017**, 53, 10006.

3. Shi, N.N.; Xie, W.J.; Zhang, D.M.; Fan, Y.H.; Cui, L.S.; Wang, M. A mononuclear copper complex as bifunctional electrocatalyst for CO_2 reduction and water oxidation. *J. Electroanal. Chem*. **2021**, 886, 115106.
4. Yin, X.M.; Zhang, S.F.; Wang, J.M.; Li, J.J.; Chen, F.F.; Yao, S.; Fan, Y.H.; Wang, M. Bioinspired cobalt molecular electrocatalyst for water oxidation coupled with carbon dioxide reduction. *Appl. Organomet. Chem*. **2021**, 35, e6371.
5. Hu, Q.Q; Su, X.J; Zhang, M.T. Electrocatalytic Water Oxidation by an Unsymmetrical Di-Copper Complex. *Inorg. Chem.* **2018**, 57, 10481-10484.
6. Makhado, T.; Das, B.; Kriek, R. J.; Vosloo, H. C. M.; Swarts, A. J. Chemical and electrochemical water oxidation mediated by bis(pyrazol-1-ylmethyl)pyridine-ligated Cu(I) complexes. *Sustain. Energ. Fuels* **2021**, 5, 2771-2780.
7. Liu, C.; Geer, Ana M.; Webber, C.; Musgrave, C.B.; et.al Immobilization of "Capping Arene" Cobalt(II) Complexes on Ordered Mesoporous Carbon for Electrocatalytic Water Oxidation. *ACS Catal.* **2021**, 11, 15068-15082.
8. Li, P.; Liu, J.B; Han, S.; Deng, W.; Yao, Z.J. Half-sandwich Ir (III) and Rh (III) complexes as catalysts for water oxidation with double-site. Appl. *Organomet. Chem.* **2019**, 33.
9. Li, X.H.; Panetier, J.A. Mechanistic Study of Tungsten Bipyridyl Tetracarbonyl Electrocatalysts for CO_2 Fixation: Exploring the Roles of Explicit Proton Sources and Substituent Effects. *Top. Catal.* **2022**, 65, 325-340.
10. Wei, S.T.; Zou, H.Y.; Rong, W.F.; Zhang, F.X.; Ji, Y.F; Duan, L.L. Conjugated nickel phthalocyanine polymer selectively catalyzes CO_2-to-CO conversion in a wide operating potential window. *Appl. Catal. B* **2021**, 284, 119739.
11. Chi, S.Y.; Chen, Q.; Zhao, S.S.; Si, D.H.; Wu, Q.J.; Huang, Y.B.; Cao, R. Three-dimensional porphyrinic covalent organic frameworks for highly efficient electroreduction of carbon dioxide. *J. Mater. Chem. A* **2022**, 10, 4653-4659.
12. Hooe, S.L.; Dressel, J.M.; Dickie, D.A.; Machan, C.W. Highly Efficient Electrocatalytic Reduction of CO_2 to CO by a Molecular Chromium Complex. *ACS Catal.* **2020**, 10, 1146-1151.
13. Ahsan, H.Md.; Breedlove, B.K.; Piangrawee, S.; Mian, M.R.; Fetoh, A.; Cosquer, G.; Yamashita, M. Enhancement of electrocatalytic abilities for reducing carbon dioxide: functionalization with a redox-active ligand-coordinated metal complex. *Dalton. T.* **2018**, 47, 11313-11316.
